# *PPARγ *Pro^12^Ala polymorphism and risk of acute coronary syndrome in a prospective study of Danes

**DOI:** 10.1186/1471-2350-10-52

**Published:** 2009-06-07

**Authors:** Ulla Vogel, Stine Segel, Claus Dethlefsen, Anne Tjønneland, Anne Thoustrup Saber, Håkan Wallin, Majken K Jensen, Erik B Schmidt, Paal Skytt Andersen, Kim Overvad

**Affiliations:** 1 National Food Institute, Technical University of Denmark, DK-2860 Søborg, Denmark and Institute of Science, Systems and Models, Roskilde University, DK-4000 Roskilde, Denmark; 2 National Research Centre for the Working Environment, DK-2100 Copenhagen, Denmark; 3 Department of Cardiology, Aalborg Hospital, Aarhus University Hospital, DK-9100 Aalborg, Denmark; 4 Center for Cardiovascular Research, Aalborg Hospital, Aarhus University Hospital, DK-9100, Aalborg, Denmark; 5 Department of Clinical Epidemiology, Aarhus University Hospital, Aalborg, DK-9100 Aalborg, Denmark; 6 Institute of Cancer Epidemiology, Danish Cancer Society, DK-2100 Copenhagen, Denmark; 7 State Serum Institut, DK-2300 Copenhagen S, Denmark

## Abstract

**Background:**

Acute coronary syndrome (ACS) is a major cause of morbidity and mortality in the western world. Peroxisome proliferator-activated receptor γ (PPARγ) plays a key role in the regulation of the energy balance, adipocyte differentiation and lipid biosynthesis. The aim was to investigate if the polymorphism *PPARγ2 *Pro^12^Ala, which encodes a less efficient transcription factor, was associated with risk of acute coronary disease and if there were interactions between this polymorphism and factors that modify PPARγ activity, such as alcohol intake, smoking, and use of non-steroidal anti-inflammatory medicine.

**Methods:**

A case-cohort study including 1031 ACS cases and a sub-cohort of 1703 persons was nested within the population-based prospective study Diet, Cancer and Health of 57,053 individuals.

**Results:**

Homozygous male variant allele carriers of *PPARγ2 *Pro^12^Ala were at higher risk of ACS (HR = 2.12, 95% CI: 1.00–4.48) than homozygous carriers of the Pro-allele. Among men, there was a statistically significant interaction between genotypes and alcohol intake such that homozygous variant allele carriers with a low alcohol intake were at higher risk of ACS (HR = 25.3, CI: 16.5–38.7) compared to homozygous common allele carriers (p for interaction < 0.0001). Overall, the association was only observed among homozygous variant allele carriers. Thus, all the observed associations were obtained in subgroups including small numbers of cases. It is therefore possible that the observed associations were due to chance.

**Conclusion:**

In the present study, there were no consistent associations between PPARγ Pro^12^Ala and risk of ACS, and no consistent interaction with alcohol, BMI, NSAID or smoking in relation to ACS.

## Background

Acute coronary syndrome (ACS) is a major cause of morbidity and mortality in the western world. Peroxisome proliferator-activated receptor γ (PPARγ) plays a key role in the regulation of the energy balance, adipocyte differentiation and lipid biosynthesis [1]. PPARγ regulates the expression of many adipose-specific genes via binding of a heterodimer of PPARγ and RXR (Retinoid × receptor) to regulatory response elements in target gene promoters [2].

The *PPARγ *Pro^12^Ala polymorphism results in a Pro to Ala amino acid substitution at codon 12 which is only present in the *PPARγ2 *isoform [2]. *PPARγ2 *is primarily expressed in adipose tissue. The polymorphism is the only commonly occurring missense polymorphism in Caucasians [2]. In vitro, the *PPARγ2 *Pro^12^Ala variant is a less active transcription factor, resulting in lower transcription levels of target genes [3-5]. There is evidence that the resulting mutant transcription factor profoundly affects the energy metabolism and energy balance linking the polymorphism to risk of diabetes mellitus [3,6]. Thus, in a meta analysis variant Ala-allele carriers were at 20% lower risk of diabetes mellitus [7]. Variant Ala-allele carriers have also been reported to have higher insulin sensitivity [2].

Diabetes and overweight are risk factors for coronary heart disease and ACS, and therefore the *PPARγ2 *Pro^12^Ala variant could be associated with lower risk of coronary heart disease and ACS. Indeed, variant allele carriers of *PPARγ2 *Pro^12^Ala have been reported to be at lower risk of myocardial infarction in some studies [8,9], but this was not confirmed in two prospective cohorts, where variant allele carriers were at higher risk of coronary heart disease than homozygous common allele carriers [10,11]. The varying results may be explained by a low number of cases and thus limited statistical power but may also be explained by different distributions of potential effect modifiers in the study populations. A number of lifestyle factors could interact with *PPAR-γ2 *Pro^12^Ala in relation to risk of ACS including alcohol intake, use of non-steroidal anti-inflammatory drugs (NSAID), anthropometry and tobacco smoking. Moreover, interaction has been reported between the *PPAR-γ2 *Pro^12^Ala polymorphism and alcohol intake in relation to plasma levels of lipoproteins [12].

We hypothesized that carriers of the *PPAR-γ2 *Pro^12^Ala polymorphism would be at lower risk of ACS and that the association was be modified by alcohol consumption, smoking, NSAID use and body mass index (BMI). We have tested the hypothesis in a case-cohort study including 1031 cases with ACS and a random cohort sample of 1703 participants from the large prospective Diet, Cancer and Health cohort, encompassing 57 053 Danes.

## Methods

### Subjects

The subjects were selected from the Danish Diet, Cancer and Health study, an ongoing prospective cohort study [13]. Between December 1993 and May 1997, 160,725 individuals aged 50 to 64 years, born in Denmark, living in the Copenhagen and Aarhus areas were invited to participate in the study. A total of 57 053 persons accepted the invitation. At enrolment, detailed information on diet, lifestyle, weight, height, reproduction status, medical treatment, and other socio-economic characteristics and environmental exposures were registered. The questionnaire is described in detail elsewhere [13]. Blood, urine, and fat tissue was sampled and stored at -150°C.

A case-cohort study was designed using incident ACS, which includes unstable angina pectoris, fatal and non-fatal myocardial infarction as the outcome [14,15]. Information on the disease endpoint was obtained by linkage with central Danish registries via the unique identification number assigned to all Danish citizens. Hospital records of potential cases were retrieved for participants who were registered with a first-time discharge diagnosis of ACS (ICD-8 codes 410–410.99, 427.27 and ICD-10 codes I20.0, I21.×, I46.×) in The Danish National Register of Patients, which covers all hospital discharge diagnoses since 1977 and all discharge diagnosis from out-patient clinics since 1995 (until Jan 1, 2004). Cases were classified according to symptoms, signs, coronary biomarkers, ECGs and/or autopsy findings in accordance with the current recommendations of the American Heart Association and the European Society of Cardiology (AHA/ECS) [14].

Further, linkage to the Cause of Death Register allowed for identification of participants with ACS coded as a primary or secondary cause of death (until Jan 1, 2004). In total, 1144 cases of ACS were identified and validated. The cohort sample included 1816 participants selected as a random sample of the entire cohort. This sample included 36 ASC cases. 183 samples were excluded because the last cases were identified after the time of sample retrieval or due to missing blood samples, and two participants were excluded due to failed genotyping. Thirteen cases and 13 controls were excluded due to lack of information on questionnaire data, leaving 1031 case subjects and 1703 participants in the sub-cohort including 34 cases who were also sub-cohort members for the analysis.

### Alcohol, NSAID, and other lifestyle variables

In the food-frequency questionnaire, alcohol intake was recorded as the average frequency of intake of six types of alcoholic beverage over the preceding year: the frequency of consumption of three strengths of beer was recorded in bottles (330 ml), wine in glasses (125 ml), fortified wine in drinks (60 ml) and spirits in drinks (30 ml). The predefined responses were in twelve categories, ranging from "never" to "eight or more times a day". The alcohol content was calculated as follows: one bottle of light beer, 8.9 g ethanol; regular beer, 12.2 g ethanol; strong beer, 17.5 g ethanol; one glass of wine, 12.2 g ethanol; one drink of fortified wine, 9.3 g ethanol; and one drink of spirits, 9.9 g ethanol.

The lifestyle questionnaire included the following question regarding use of NSAID: Have you taken more than one pain relieving pill per month during the last year? If the answer was yes, the participant was asked to record how frequent they took each of the following types of medications: "Aspirin", "Paracetamol", "Ibuprofen", or "Other pain relievers". The latter category included NSAID preparations other than aspirin and ibuprofen. Based on all records, we classified study subjects according to their use of "any NSAID" (≥ 2 pills per month during one year) at baseline.

Data on hormone replacement therapy (HRT) was obtained from the lifestyle questionnaire and the participants were classified according to use HRT (never, past or current). Smoking status was recorded in three categories: never smoker, current smoker and former smoker.

At the study clinics, anthropometric measurements, including height and weight were obtained by professional staff members. BMI was calculated as weight (kg) per height squared (m).

### Blood sampling and storage

From each non-fasting participant a total of 30 ml blood was collected in citrated (2 × 10 ml) and plain (1 × 10 ml) Venojects. Plasma, serum, lymphocytes, and erythrocytes were isolated and frozen at -20°C within 2 hours. At the end of the day of collection, all samples were stored in liquid nitrogen at -150°C.

### Genotyping

DNA was isolated from frozen lymphocytes as described by Miller et al [16]. Generally, 100 μg DNA were obtained from 10^7 ^lymphocytes. *PPAR2γ *Pro^12^Ala (rs1801282) was genotyped as previously described [17]. Laboratory staff was blinded to the case/subcohort status of the subjects. Twenty ng of DNA were used for genotyping in 5 μl containing 1× Mastermix (Applied Biosystems, Nærum, Denmark), 100 nM probes, and 900 nM primers. Controls were included in each run, and repeated genotyping of a random 10% subset yielded 100% identical genotypes.

### Measurement of blood lipids

Total Cholesterol and other blood lipids were measured on an Advia 1650 from Bayer Diagnostics, NY, USA. For total cholesterol kits ref. 01482198 were used. The interseriel variation was 1.1%. For triglyceride kits ref. 09580156 was used with an interserial variation of 4.5%. For HDL-cholesterol kits ref. 08058065 with an interseriel variation of 2.0%. LDL-cholesterol was calculated by Friedewald's formula.

### Statistical methods

We used a case-cohort design with a sub-cohort of 1703 subjects drawn randomly, stratified on gender from the whole cohort with 57,053 subjects. A Cox proportional hazards model was used for analyses, as if the full cohort were included, modified by a weighting scheme described [18] and using a robust variance estimate. In the case-cohort design, weights are assigned to each subject, one for cases and N/n for non-cases in the sub-cohort, where N (n) is the number of non-cases in the cohort (sub-cohort). For women, N/n = 29,289/797 and for men, N/n = 26,012/906.

Age was used as the time scale in the Cox regression model. All models were sex specific and adjusted for baseline values of established risk factors for ACS including status of BMI, HRT, smoking status, NSAID use and alcohol intake.

We investigated possible interactions between polymorphism and alcohol intake, NSAID use, smoking status and BMI using the likelihood ratio test. Analyses were done using Stata version 9.2 (Stata Corporation, College Station, Texas, US).

### Approval

Diet, Cancer and Health and the present sub-study were approved by the regional Ethics Committees on Human Studies in Copenhagen and Aarhus (File nos.(KF)11-037/01 and HKF-01-345/93), and by the Danish Data Protection Agency.

## Results

The final case group consisted of 786 men and 245 women who were diagnosed with acute coronary syndrome after recruitment into the Diet, Cancer and Health cohort and the final random cohort sample 906 men and 797 women selected from the cohort for whom genotypes and information on lifestyle variables were available. Cases and the cohort sample are described in Table 1. Both male and female cases had a higher BMI, were more likely to have diabetes mellitus, hypercholesterolemia, and hypertension, be smokers and have a lower alcohol intake than the cohort sample. They also had higher triglyceride levels and lower HDL levels in plasma.

**Table 1:** Baseline characteristics of the study participants.

	**Women**		**Men**	
**Variable^a^**	**Cases** (n = 245)	**Cohort sample** (n = 797)	**Cases** (n = 786)	**Cohort sample** (n = 906)
Age at entry (yrs)	60 (52–65)	56 (51–64)	58 (51–65)	56 (51–64)
				
BMI (kg/m^2^)	26.4 (20.1–35.2)	24.6 (19.7–33.7)	26.9 (22.4–34.1)	26.3 (21.6–32.4)
				
Waist (cm)	86 (69–107)	80 (67–105)	97 (83–117)	95 (82–113)
				
Diabetes^b^				
Yes	5.3%	1.0%	5.4%	2.3%
No	90.2%	94.3%	88.5%	91.7%
Unknown	4.5%	4.7%	6.1%	6.0%
				
Hypercholesterolemia^b^				
Yes	16.9%	6.1%	12.1%	9.7%
No	44.0%	48.4%	45.4%	52.0%
Unknown	39.1%	45.5%	42.5%	38.3%
				
Hypertension				
Yes	40.0%	15.5%	22.2%	13.1%
No	51.8%	74.9%	61.0%	72.4%
Unknown	8.2%	9.6%	16.8%	14.5%
				
Postmenopausal				
Yes	70.2%	57.8%	N/A	N/A
No	5.3%	17.5%		
Unknown	24.5%	24.7%		
				
HRT use				
Current	26.5%	31.7%	N/A	N/A
Former	22.5%	15.7%		
Never	51.0%	52.6%		
				
Smoking				
Current	59.2%	36.8%	59.3%	37.3%
Former	16.3%	21.4%	26.5%	35.4%
Never	24.5%	41.8%	14.2%	27.3%
				
Alcohol (g/d)	6 (0–38)	9 (0–40)	17 (1–72)	20 (1–83)
				
Plasma lipids (mmol/L)				
				
Triglycerides	1.8 (0.9–4.7)	1.3 (0.7–3.2)	2.1 (0.9–5.3)	1.7 (0.8–4.4)
				
Cholesterol	6.5 (4.8–8.6)	6.0 (4.5–7.9)	6.3 (4.6–8.1)	5.9 (4.6–7.7)
				
HDL-C	1.6 (1.1–2.2)	1.8 (1.2–2.6)	1.3 (1.0–1.9)	1.4 (1.0–2.1)

a) Medians (5th and 95th percentiles) of continuous covariates. All lipids were obtained from non-fasting participants (n = 2610) b) Diagnosed with hypercholesterolemia.

The genotype distribution of *PPARγ *Pro^12^Ala was in Hardy-Weinberg equilibrium for both men and women in the cohort sample (results not shown). The allele frequency of the variant allele was 0.130 among men and 0.141 among women in the random sample. This is similar to the 0.14 found previously for another sample of the DHC cohort of 317 men and women matched on age and sex to 317 basal cell carcinoma cases [19].

Among men, homozygous carriers of the variant allele of *PPARγ *Pro^12^Ala were at higher risk of acute coronary syndrome than homozygous common allele carriers (hazard ratio (HR) = 2.12, 95% confidence interval (CI): 1.00–4.48) (Table 2). In an additive model, the polymorphism was not statistically significantly associated with risk of ACS (p = 0.70). A negative though statistically insignificant association was observed among women. The HR for female homozygous carriers of the variant allele of *PPARγ *Pro^12^Ala was 0.40 (0.08–1.85). In an additive model, the polymorphism was not statistically significantly associated with risk of ACS (p = 0.35).

**Table 2:** Association between PPARγ Pro^12^Ala and risk of acute coronary syndrome among men and women in the Diet, Cancer and Health Cohort

*PPARγ *Pro^12^Ala	N_case_/N_non-cases_	HR^a^ (95% CI)	p	HR^b^ (95% CI)	p	HR^c^ (95% CI)	p
Men							
Pro/Pro	589/664	1.0		1.0			
Pro/Ala	176/204	0.92 (0.73–1.17)	0.51	0.92 (0.72–1.18)	0.50	Not relevant	
Ala/Ala	21/12	1.99 (0.98–4.05)	0.06	2.12 (1.00–4.48)	0.05	Not relevant	
Pro/Ala & Ala/Ala	197/216	0.98 (0.78–1.23)	0.86	0.98 (0.77–1.24)	0.86	Not relevant	
Women							
Pro/Pro	181/581	1.0		1.0		1.0	
Pro/Ala	62/193	0.96 (0.67–1.36)	0.80	0.93 (0.65–1.35)	0.75	0.96 (0.67–1.40)	0.85
Ala/Ala	2/15	0.38 (0.08–1.72)	0.21	0.37 (0.08–1.72)	0.21	0.40 (0.08–1.85)	0.24
Pro/Ala & Ala/Ala	64/208	0.91 (0.65–1.29)	0.60	0.89 (0.62–1.27)	0.53	0.92 (0.64–1.33)	0.60

a) Crude (only adjusted for age)

b) Adjusted for alcohol, smoking and NSAID

c) Adjusted for alcohol, smoking, NSAID, hormone replacement therapy and menopause status

To investigate a possible interaction between *PPARγ *Pro^12^Ala and alcohol intake, alcohol intake was subdivided into tertiles according to the distribution of intake among women in the cohort sample. 4 g/day and 12 g/day were used as cut points among both men and women (Table 3). For men, there was interaction between *PPARγ *Pro^12^Ala and alcohol intake. The effect was primarily carried by a high risk of ACS among homozygous variant allele carriers who drank less than 4 gram alcohol/day (HR = 25.3, CI: 16.5–38.7), which was not present among those with a higher alcohol consumption. There was no interaction between *PPARγ *Pro^12^Ala and alcohol consumption among women.

**Table 3:** HR for acute coronary syndrome subdivided by alcohol intake and PPARγ Pro^12^Ala genotype.

	N_cases_/N_non-cases_			HR (95% CI)		
	Alcohol intake g/day			Alcohol intake g/day		
*PPARγ *Pro^12^Ala	< 4.0	4.0 < × < 12.0	> 12.0	< 4.0	4.0 < × < 12.0	> 12.0
Men						
Pro/Pro	98/81	127/128	364/455	1^a^	0.86 (0.59–1.28)	0.70 (0.50–0.98)
Pro/Ala	30/19	41/37	105/148	1.41 (0.73–2.70)	0.90 (0.64–1.55)	0.58 (0.39–0.86)
Ala/Ala	4/0	4/1	13/11	40.7 (27.5–60.2)	2.30 (0.29–18.5)	1.09 (0.46–2.57)
P for interaction						p < 0.0001
Pro/Pro	98/81	127/128	364/455	1^b^	0.91 (0.60–1.37)	0.69 (0.49–0.99)
Pro/Ala	30/19	41/37	105/148	1.30 (0.67–2.55)	0.78 (0.44–1.39)	0.60 (0.39–0.90)
Ala/Ala	4/0	4/1	13/11	25.3 (16.5–38.7)	3.18 (0.41–24.8)	1.11 (0.45–2.76)
P for interaction						p < 0.0001
Women						
Pro/Pro	76/157	50/205	55/219	1^a^	0.52 (0.34–0.80)	0.52 (0.34–0.79)
Pro/Ala	25/60	21/63	16/70	0.72 (0.41–1.27)	0.70 (0.39–1.27)	0.45 (0.23–0.88)
Ala/Ala	1/7	0/4	1/4	0.25 (0.29–2.15)	No estimate	0.70 (0.08–6.50)
P for interaction						0.34
Pro/Pro	76/157	50/205	55/219	1^b^	0.53 (0.34–0.83)	0.51 (0.33–0.80)
Pro/Ala	25/60	21/63	16/70	0.73 (0.40–1.31)	0.71 (0.38–1.31)	0.44 (0.22–0.88)
Ala/Ala	1/7	0/4	1/4	0.27 (0.03–2.15)	No estimate	0.52 (0.05–5.30)
P for interaction						0.46
Pro/Pro	76/157	50/205	55/219	1^c^	0.53 (0.34–0.84)	0.52 (0.33–0.82)
Pro/Ala	25/60	21/63	16/70	0.75 (0.41–1.35)	0.72 (0.38–1.35)	0.48 (0.24–0.86)
Ala/Ala	1/7	0/4	1/4	0.29 (0.03–2.59)	No estimate	0.55 (0.05–5.71)
P for interaction						0.51

a) Crude analysis (age adjusted), b) Adjusted for smoking and alcohol intake, c) Adjusted for smoking, alcohol, menopause status and HRT use

There was no interaction between *PPARγ *Pro^12^Ala and BMI neither among men nor women (results not shown).

There was no interaction between *PPARγ *Pro^12^Ala and NSAID use in relation to ACS, neither when aspirin use was included in the group of 'no NSAID use' (table 4) nor when aspirin use was included as a separate category (results not shown).

**Table 4:** Risk of acute coronary syndrome in relation to use of NSAID. Aspirin users are included in 'No NSAID'

***PPARγ *Pro^12^Ala**	**N_cases_/N_non-cases_**		**HR^a ^95% CI**				**HR^b ^95% CI**			
Men	NSAID		NSAID				NSAID			
	NO	YES	NO		YES		NO		YES	
Pro/Pro	531/622	58/42	1.00	-	1.63	1.07–2.47	1.00	-	1.58	1.00–2.48
Pro/Ala	156/190	20/14	0.91	0.75–1.17	1.61	0.79–3.25	0.91	0.70–1.17	1.63	0.79–3.37
Ala/Ala	20/11	1/1	2.07	0.99–4.32	2.00	0.13–30.5	2.36	1.09–5.13	1.10	0.07–17.7
P for interaction						0.918				0.670
										
Pro/Pro	531/622	58/42	1.00	-	1.63	1.07–2.47	1.00	-	1.58	1.00–2.48
Pro/Ala & Ala/Ala	176/201	21/15	0.96	0.77–1.24	1.62	0.72–3.23	0.97	0.76–1.25	1.60	0.79–3.23
P for interaction						0.958				0.929
Women										
Pro/Pro	145/505	36/76	1.00	-	1.52	0.96–2.41	1.00	-	1.37	0.84–2.23
Pro/Ala	52/168	10/25	0.99	0.67–1.45	1.31	0.59–2.87	0.93	0.62–1.38	1.33	0.59–3.00
Ala/Ala	2/9	0/6	0.61	0.12–2.97	-	-	0.54	0.11–2.71	-	-
P for interaction						0.771				0.924
										
Pro/Pro	145/505	36/76	1.00	-	1.42	0.96–2.41	1.00	-	1.36	0.84–2.23
Pro/Ala & Ala/Ala	54/177	10/31	0.96	0.66–1.41	1.08	0.50–2.32	0.90	0.61–1.34	1.14	0.52–2.51
P for interaction						0.517				0.875

a) crude

b) adjusted for smoking status and alcohol consumption

Male smokers were at almost 3-fold higher risk of ASC than non-smokers (additional file 1). There was no interaction between *PPARγ *Pro^12^Ala and smoking for men. Among women, there was interaction between *PPARγ *Pro^12^Ala and smoking status (p = 0.001). Variant allele carriers were at lower risk of ASC among never smokers and past smokers. There was no association between cholesterol levels and *PPARγ *Pro^12^Ala, and no interaction between *PPARγ *Pro^12^Ala and alcohol intake in relation to cholesterol levels or triglyceride levels (additional file 2).

## Discussion

In this rather large, prospective study of the association between *PPARγ *Pro^12^Ala and risk of ACS, there was very little effect of the *PPARγ *Pro^12^Ala polymorphism. Homozygous variant allele carriers were at higher risk among men whereas the variant allele was associated with lower risk among women. The associations were only observed among homozygous carriers of the variant allele. Potential effect modification from intake of alcohol, use of NSAID, anthropometric variables and tobacco smoking was explored, but no consistent interactions were observed. All significant associations were observed in subgroups with small numbers of cases and limited power, indicating that the findings may be due to chance. The results are in agreement with a recent meta-analysis finding no association [20].

Our cases and controls were selected from the same cohort, which together with an almost complete follow-up of the participants minimized the risk for selection bias. For all participants, information on lifestyle factors was collected at enrolment, which minimized the risk for differential misclassification between the cases and controls. Furthermore, the use of only ACS cases confirmed through medical records ensured a high validity of our endpoint data.

We calculated separate risk estimates for men and women. This is partly because the risk of acute coronary heart disease is very different between the sexes, partly because some of the previous investigations were made among men only [9]. Also the prevalence of potential effect modifiers varied between the sexes in the present cohort. It has previously been reported that *PPARγ *Pro^12^Ala interact with the closely linked polymorphism *PPARγ *C14314T [8,21,22]. In the present cohort of Danes, the two polymorphisms are closely linked, with 84% of the genotypes being concordant and only 16% discordant [23]. We therefore made no attempt to differentiate between the effects of the two polymorphisms.

Among men, there was a statistically significant interaction between *PPARγ *Pro^12^Ala and alcohol intake. Among those who drank alcohol, variant allele carriers were at higher risk of ACS at all levels of alcohol intake but the association was strongest among participants with the lowest alcohol intake where heterozygous carriers were at 1.3-fold higher risk (95%CI: 0.67–2.55) of ACS than homozygous common allele carriers and homozygous variant allele carriers at 25.3 (95%CI: 16.5–38.7) times higher risk of ACS. These results are consistent with another prospective study [10], where male carriers of the variant allele were at 1.44-fold (95%CI: 1.00–2.07) higher risk than homozygous common allele carriers in a cohort where the average alcohol consumption among men was 7.0 g/day among controls and 5.9 g/day among cases. Information on alcohol consumption was not included in other studies [9,24]. However, the present findings among those with a low alcohol intake rely on few cases, and among women no consistent effect modification was observed.

We were unable to reproduce a previously found interaction between *PPARγ *Pro^12^Ala and overweight in relation to coronary heart disease, where it was found that the variant allele was a stronger risk factor among overweight men and women [10]. We observed no interaction between *PPARγ *Pro^12^Ala and blood levels of cholesterol, or triglycerides, and no interaction with alcohol. Thus, we were also unable to reproduce a previously found interaction [12]. We have almost used the same cut-off value (4 g/day compared to 5 g/day), but the other study [12] included much younger individuals with lower cholesterol and triglyceride blood levels. Furthermore, the previous study did not subdivide by sex, although cholesterol levels differ by sex. Studies of mice, which were specifically deleted for the *PPARγ2 *isoform of *PPARγ *had the same bodyweight as common allele littermates when fed chow, but developed less adipose tissue when put on a high fat diet [25]. Thus, it is possible that the lack of associations for the *PPARγ *Pro^12^Ala is because of the relatively low fat diets among the present cohort members.

A number of commonly used NSAIDs activate PPARγ [26], and thus, if differences in PPARγ activity is related to risk of ACS, then interaction with NSAID use would be expected. We have previously observed interaction between *PPARγ *Pro^12^Ala and NSAID use in relation to risk of both breast cancer and lung cancer [17,27] using the Diet, Cancer and Health cohort and the same definitions of NSAIDs as used here. No interaction between *PPARγ *Pro^12^Ala and NSAID use in relation to risk of ACS was, however, observed in this study. This indicates either that PPARγ activity is not related to risk of ACS, or that the activation of target genes in relation to risk of ACS is different than for cancer.

Among women, there was interaction between smoking status and *PPARγ *Pro^12^Ala. Variant allele carriers were at lower risk of ACS than homozygous common allele carriers among never and past smokers. We have previously observed the same pattern in relation to lung cancer, although there was no statistically significant interaction [27]. Among men, there seemed to be only additive effects of *PPARγ *Pro^12^Ala and smoking. Since the interaction among women relied on relatively few persons among non-smokers and variant allele carriers, the interaction observed may be a chance finding.

## Conclusion

In conclusion, there were no convincing associations between *PPARγ *Pro^12^Ala and risk of ACS, and no systematic interaction with alcohol, BMI, NSAID or smoking in relation to ACS.

## Competing interests

The authors declare that they have no competing interests.

## Authors' contributions

UV, KO, PSA and ATS conceived the study. AT and KO established the 'Diet, Cancer and Health' study. UV and HWA isolated DNA and determined the polymorphism. EBS determined the blood lipids. UV, KO, PSA and SS designed the statistical analyses. SS performed the statistical analyses. All authors participated in the analysis and interpretation of the data. UV drafted the manuscript and all authors read and approved the final version of the manuscript.

## Pre-publication history

The pre-publication history for this paper can be accessed here:



## Supplementary Material

Additional file 1 **Table 5**. Word file containing table 5 landscape format. Click here for file

Additional file 2 **Table 6**. Word file containing table 6 landscape format. Click here for file
