# Supplementary material for: PPARγ Pro12Ala polymorphism and risk of acute coronary syndrome in a prospective study of Danes
Source: BMC Med Genet. 2009 Jun 7;10:52. doi: 10.1186/1471-2350-10-52 (PMC2698834; doi:10.1186/1471-2350-10-52)
Supplement: Additional file 1 — Table 5. Word file containing table 5 landscape format. [file 1471-2350-10-52-S1.doc]

*Table 5. Risk of acute coronary syndrome in relation to PPARγ Pro^12^Ala genotype and smoking status.*

| ***PPARγ* Pro^12^Ala** | **N_cases_/N_non-cases_** | | | **HR (95% CI)^a^** | | | | | | **HR (95% CI)^b^** | | | | | |
| --- | --- | --- | --- | --- | --- | --- | --- | --- | --- | --- | --- | --- | --- | --- | --- |
|  | Smoking status | | | Smoking status | | | | | | Smoking status | | | | | |
| MEN | Never | Past | Current | Never | | Past | | Current | | Never | | Past | | Current | |
| Pro/Pro | 82/192 | 163/229 | 344/243 | 1.00 | - | 1.55 | 1.11-2.15 | 3.07 | 2.25-4.19 | 1.00 | - | 1.57 | 1.12-2.19 | 3.15 | 2.31-4.32 |
| Pro/Ala | 26/45 | 41/77 | 109/82 | 1.25 | 0.72-2.17 | 1.11 | 0.70-1.76 | 2.81 | 1.90-4.17 | 1.27 | 0.73-2.21 | 1.15 | 0.72-1.82 | 2.89 | 1.93-4.30 |
| Ala/Ala | 4/3 | 4/6 | 13/3 | 3.41 | 0.76-15.21 | 1.38 | 0.38-5.04 | 9.80 | 2.91-32.90 | 4.02 | 0.90-17.91 | 1.38 | 0.38-5.04 | 9.74 | 2.93-32.31 |
| p_int_ |  |  |  |  |  |  |  |  | 0.32 |  |  |  |  |  | 0.31 |
| WOMEN |  |  |  |  |  |  |  |  |  |  |  |  |  |  |  |
| Pro/Pro | 50/242 | 31/131 | 100/208 | 1.00 | - | 1.03 | 0.61-1.73 | 2.20 | 1.47-3.30 | 1.00 | - | 1.04 | 0.62-1.76 | 2.18 | 1.45-3.29 |
| Pro/Ala | 10/82 | 8/34 | 44/77 | 0.53 | 0.25-1.11 | 0.96 | 0.41-2.24 | 2.56 | 1.53-4.28 | 0.51 | 0.24-1.07 | 0.94 | 0.40-2.21 | 2.58 | 1.54-4.32 |
| Ala/Ala | 0/6 | 1/4 | 1/5 | - | - | 0.96 | 0.09-9.77 | 0.74 | 0.08-6.63 | - | - | 0.79 | 0.08-8.21 | 0.75 | 0.08-6.76 |
| p_int_ |  |  |  |  |  |  |  |  | 0.001 |  |  |  |  |  | 0.001 |

1. Crude (age adjusted).
2. In addition, adjusted for NSAID and alcohol consumption.
