# Supplementary material for: PPARγ Pro12Ala polymorphism and risk of acute coronary syndrome in a prospective study of Danes
Source: BMC Med Genet. 2009 Jun 7;10:52. doi: 10.1186/1471-2350-10-52 (PMC2698834; doi:10.1186/1471-2350-10-52)
Supplement: Additional file 2 — Table 6. Word file containing table 6 landscape format. [file 1471-2350-10-52-S2.doc]

*Table 6. LDL, HDL, total cholesterol and triglyceride levels subdivided by sex, PPARγ Pro^12^Ala genotype and alcohol intake.*

| ***PPAR-γ***  ** P^12^A** | **LDL-levels (95%CI)** | | **HDL-levels (95%CI)** | | **Total cholesterol levels (95%CI)** | | **Triclyceride levels (95%CI)** | |
| --- | --- | --- | --- | --- | --- | --- | --- | --- |
|  | Alkohol intake | | Alcohol intake | | Alcohol intake | | Alcohol intake | |
|  |  |  |  |  |  |  |  |  |
| Men: | <4 g/d | >4 g/d | <4 g/d | >4 g/d | <4 g/d | >4 g/d | <4 g/d | >4 g/d |
| Pro/Pro | 3.89 (3.76-4.03) | 3.75 (3.70-3.81) | 1.31 (1.26-1.36) | 1.43 (1.41-1.45) | 6.11 (5.96-6.27) | 6.10 (6.04-6.16) | 2.00 (1.86-2.15) | 2.02 (1.96-2.08) |
| Pro/Ala | 3.73 (3.48-3.99) | 3.71 (3.61-3.81) | 1.25 (1.15-1.34) | 1.43 (1.40-1.47) | 5.82 (5.54-6.10) | 6.06 (5.96-6.17) | 1.84 (1.57-2.11) | 2.03 (1.92-2.13) |
| Ala/Ala | 4.31 (3.30-5.32) | 3.66 (3.34-3.97) | 1.18 (0.88-1.56) | 1.44 (1.32-1.56) | 6.26 (5.16-7.37) | 6.01 (5.66-6.37) | 1.69 (0.62-2.75) | 2.02 (1.68-2.35) |
| p ^a^ |  | 0.47 |  | 0.39 |  | 0.29 |  | 0.52 |
| p ^b^ |  | 0.53 |  | 0.98 |  | 0.47 |  | 0.95 |
| Women |  |  |  |  |  |  |  |  |
|  | <4 g/d | >4 g/d | <4 g/d | >4 g/d | <4 g/d | >4 g/d | <4 g/d | >4 g/d |
| Pro/Pro | 3.83 (3.70-3.95) | 3.58 (3.49-3.66) | 1.65 (1.60-1.71) | 1.84 (1.80-1.88) | 6.23 (6.09-6.37) | 6.13 (6.04-6.22) | 1.66 (1.55-1.76) | 1.58 (1.50-1.65) |
| Pro/Ala | 3.78 (3.57-3.99) | 3.66 (3.51-3.80) | 1.63 (1.54-1.72) | 1.78 (1.72-1.85) | 6.17 (5.94-6.40) | 6.16 (6.00-6.33) | 1.68 (1.50-1.85) | 1.60 (1.47-1.72) |
| Ala/Ala | 3.42 (2.75-4.09) | 3.17 (2.58-3.77) | 1.54 (1.24-1.83) | 1.76 (1.50-2.03) | 5.75 (5.01-6.50) | 5.94 (5.28-6.60) | 1.74 (1.18-2.30) | 2.21 (1.71-2.71) |
| p ^a^ |  | 0.69 |  | 0.87 |  | 0.74 |  | 0.36 |
| p ^b^ |  | 0.18 |  | 0.24 |  | 0.46 |  | 0.13 |

a) p-value for interaction between PPARγ and alcohol on the lipid level

b) p-value for effect of PPARγ on the lipid level. Allows for different alcohol-levels.
